# Umbilical cord blood DNA methylation in children who later develop type 1 diabetes

**DOI:** 10.1007/s00125-022-05726-1

**Published:** 2022-06-18

**Authors:** Essi Laajala, Ubaid Ullah Kalim, Toni Grönroos, Omid Rasool, Viivi Halla-aho, Mikko Konki, Roosa Kattelus, Juha Mykkänen, Mirja Nurmio, Mari Vähä-Mäkilä, Henna Kallionpää, Niina Lietzén, Bishwa R. Ghimire, Asta Laiho, Heikki Hyöty, Laura L. Elo, Jorma Ilonen, Mikael Knip, Riikka J. Lund, Matej Orešič, Riitta Veijola, Harri Lähdesmäki, Jorma Toppari, Riitta Lahesmaa

**Affiliations:** 1 Turku Bioscience Centre, University of Turku and Åbo Akademi University, Turku, Finland; 2 InFLAMES Research Flagship Center, University of Turku, Turku, Finland; 3 Turku Doctoral Programme of Molecular Medicine, University of Turku, Turku, Finland; 4 Department of Computer Science, Aalto University, Espoo, Finland; 5 Research Centre of Applied and Preventive Cardiovascular Medicine, University of Turku, Turku, Finland; 6 Centre for Population Health Research, University of Turku and Turku University Hospital, Turku, Finland; 7 Research Centre for Integrative Physiology and Pharmacology, Institute of Biomedicine, University of Turku, Turku, Finland; 8 Institute for Molecular Medicine Finland (FIMM), University of Helsinki, Helsinki, Finland; 9 Department of Virology, Faculty of Medicine and Biosciences, University of Tampere, Tampere, Finland; 10 Institute of Biomedicine, University of Turku, Turku, Finland; 11 Immunogenetics Laboratory, Institute of Biomedicine, University of Turku, Turku, Finland; 12 Pediatric Research Center, Children’s Hospital, University of Helsinki and Helsinki University Hospital, Helsinki, Finland; 13 Research Program for Clinical and Molecular Metabolism, Faculty of Medicine, University of Helsinki, Helsinki, Finland; 14 Center for Child Health Research, Tampere University Hospital, Tampere, Finland; 15 School of Medical Sciences, Örebro University, Örebro, Sweden; 16 Department of Pediatrics, PEDEGO Research Unit, Medical Research Center, Oulu University Hospital and University of Oulu, Oulu, Finland; 17 Department of Pediatrics, Turku University Hospital, Turku, Finland

**Keywords:** Bisulphite sequencing, DNA methylation, Epigenomics, Follow-up study, Type 1 diabetes, Umbilical cord blood

## Abstract

**Aims/hypothesis:**

Distinct DNA methylation patterns have recently been observed to precede type 1 diabetes in whole blood collected from young children. Our aim was to determine whether perinatal DNA methylation is associated with later progression to type 1 diabetes.

**Methods:**

Reduced representation bisulphite sequencing (RRBS) analysis was performed on umbilical cord blood samples collected within the Finnish Type 1 Diabetes Prediction and Prevention (DIPP) Study. Children later diagnosed with type 1 diabetes and/or who tested positive for multiple islet autoantibodies (*n* = 43) were compared with control individuals (*n* = 79) who remained autoantibody-negative throughout the DIPP follow-up until 15 years of age. Potential confounding factors related to the pregnancy and the mother were included in the analysis.

**Results:**

No differences in the umbilical cord blood methylation patterns were observed between the cases and controls at a false discovery rate <0.05.

**Conclusions/interpretation:**

Based on our results, differences between children who progress to type 1 diabetes and those who remain healthy throughout childhood are not yet present in the perinatal DNA methylome. However, we cannot exclude the possibility that such differences would be found in a larger dataset.

**Graphical abstract:**

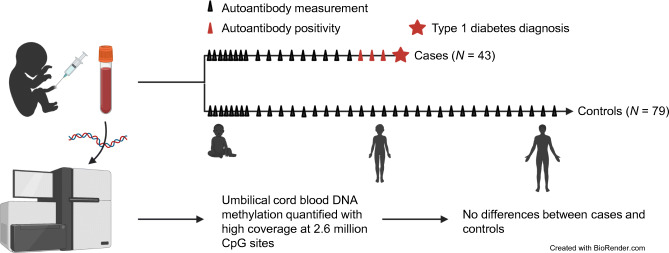

**Supplementary Information:**

The online version contains peer-reviewed but unedited supplementary material available at 10.1007/s00125-022-05726-1.

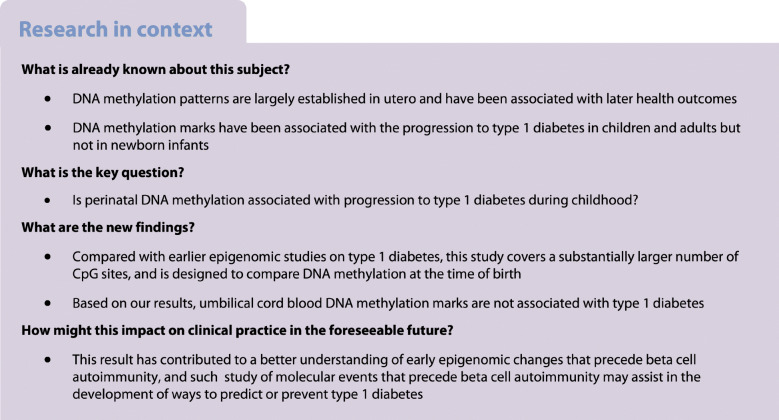



## Introduction

DNA methylation at cytosine residues is one of the most important epigenetic mechanisms regulating gene expression. The modification converts cytosine to 5-methylcytosine, usually in the context of CpG dinucleotides. Differential methylation at the promoter or other regulatory elements affects gene expression in health and diseases [1]. Most studies on the association between type 1 diabetes and DNA methylation have focused on differences between case and control participants at the time of diagnosis or later. The most extensive study on the topic included immune effector cells from 52 monozygotic twin pairs who were discordant for type 1 diabetes [2]. Thousands of CpG sites were found to be differentially variable between affected participants and their healthy co-twins. An earlier study [3] included a small set of samples from prediabetic individuals (*n* = 7) to confirm findings from already diagnosed participants. A recent report from the Diabetes Autoimmunity Study in the Young (DAISY) on prospective epigenomics of type 1 diabetes also included samples collected before seroconversion of the case participants to islet autoantibody positivity [4].

A peak in the appearance of islet autoimmunity occurs at an early age, between one and two years [5]. We therefore hypothesised that progression to type 1 diabetes during childhood may already be reflected in the epigenome at birth. Two previous studies have examined umbilical cord blood samples from neonates who later progressed to type 1 diabetes [2, 4]. However, in both studies, the neonatal samples were only used to confirm the direction of change in differentially methylated regions discovered at later time points. They did not publish umbilical cord blood DNA methylation measurements outside the candidate regions.

In this study we used the reduced representation bisulphite sequencing (RRBS) method to analyse umbilical cord blood DNA methylation associated with later progression to type 1 diabetes in a prospective cohort from the Finnish Type 1 Diabetes Prediction and Prevention (DIPP) Study. The aim was to detect DNA methylation patterns associated with later progression to type 1 diabetes. Such findings would be valuable for a better understanding of early mechanisms underlying the progression to type 1 diabetes related autoimmunity.

## Methods

The electronic supplementary material (ESM) provides further information regarding the power analysis (ESM Methods and ESM Fig. 1) and a more detailed description of the HLA risk class determination, sample collection, islet autoantibody measurement, RRBS, bisulphite pyrosequencing protocols and data analysis (ESM Methods and ESM Tables 1–4).

### Study design

Case participants (*n* = 43) who were diagnosed with type 1 diabetes during the DIPP Study follow-up or became persistently positive for at least two biochemical islet autoantibodies (in at least two consecutive serum samples) were compared with control participants (*n* = 79) who remained autoantibody-negative throughout the DIPP Study follow-up, i.e. up to 15 years of age or until their decision to discontinue participation in the study. Data until the end of year 2018 were included. Clinical data such as maternal insulin-treated diabetes, gestational weight gain and the child’s birthweight were used to adjust for potential confounding effects. The characteristics of the case and control participants are described in Table 1.

**Table 1:** Characteristics of the case and control participants

Variable	Cases (*n* = 43)	Controls (*n* = 79)	Number of missing values
Child			
Age at diagnosis of type 1 diabetes (*n* = 34) (years); median (range)	8.7 (1.6–18.8)	NA	–
Age at seroconversion (years); median (range)	2.5 (0.5–10.7)	NA	–
First biochemical autoantibody (*n*)			–
IAA	14	NA	
GADA	13	NA	
IA-2A	3	NA	
Multiple/unknown	13	NA	
HLA risk (*n*)			0
High	21	24	
Moderate	19	27	
Neutral/slightly elevated	3	28	
Sex (*n*)			0
Female	17	25	
Male	26	54	
Birthweight (g); median (range)	3750 (2310–4600)	3500 (1910–4860)	0
Apgar points at 1 min (*n*)			1
Normal (8–10)	37	68	
Low (4–7)	5	11	
Mother			
Maternal age (years); median (range)	29.8 (21.3–39.6)	30.7 (21.3–45.8)	0
Maternal height (cm); median (range)	168 (152–179)	165 (150–179)	0
Maternal BMI (before this pregnancy) (kg/m^2^); median (range)	22.7 (17.2–41.7)	23.2 (18.0–35.5)	2
Number of earlier miscarriages (*n*)			0
None	34	64	
One or more	9	15	
Pregnancy			
Gestational weight gain (mother) (kg); median (range)	13 (0–22)	14 (0–28)	3
Maternal insulin treatment for diabetes (gestational or other) (*n*)			0
Yes	4	1	
No	39	78	
Maternal smoking during pregnancy (*n*)			3
Yes	3	5	
No	39	72	
Delivery			
Mode of delivery (*n*)			0
Caesarean section	3	13	
Vaginal	40	66	
Labour induction (*n*)			0
Yes	7	14	
No	36	65	
Usage of epidural anaesthetic during delivery phase I (*n*)			0
Yes	18	40	
No	25	39	
Technical			
Year of birth; median (range)	2001 (1995–2006)	1999 (1995–2006)	0
Month of birth (*n*)			0
Dec–Feb	11	20	
Mar–May	12	23	
Jun–Aug	11	18	
Sep–Nov	9	18	
Library preparation batch (*n*)			0
1A	5	11	
1B	2	2	
1C	6	9	
2A	6	15	
2B	11	11	
3A	4	8	
3B	9	23	

Data are *n* or median (range)

All the covariates listed here were included as explanatory variables in the differential methylation analysis, except for age at diagnosis, age at seroconversion and first-appearing autoantibody, which are relevant only for the case group. The inclusion criteria are specified in ESM Table 1

Umbilical cord blood samples were collected from newborn children born in Turku University Hospital between 1995 and 2006. After informed consent, HLA DR/DQ genotyping was performed from umbilical cord blood to identify children at increased risk of developing type 1 diabetes. Eligible children were invited to participate in the DIPP Study follow-up, during which islet autoantibodies were measured 1–4 times per year using specific radio-binding assays. The islet autoantibodies included IAA (insulin autoantibody), IA-2A (insulinoma-associated protein 2 antibody), GADA (glutamic acid decarboxylase antibody) and ZnT8A (zinc transporter-8 antibody). Screening for classical islet cell antibodies was used as the only autoantibody screening method for children in the DIPP Study born before 2003, and, if positive, all other autoantibodies were measured from all previous and future samples from the child.

### Power analysis

The power analysis was performed on simulated bisulphite sequencing data using a tool developed by Lea et al [6] (see ESM Methods for details).

### Sample collection and HLA risk class determination

Umbilical cord blood was collected immediately after birth in 3 ml K3-EDTA tubes in the delivery room at Turku University Hospital. HLA DR/DQ genotypes were determined from the DNA in the dried blood spots using assays that were designed to densely probe the genomic regions associated with type 1 diabetes. The genotyping was started from major DQB1 alleles (see ESM Methods for details).

### Islet autoantibody measurement

Islet autoantibodies in serum samples were measured using specific radio-binding assays (see ESM Methods for details).

### Sample inclusion criteria

Of 200 cord blood samples, 20 were excluded for low (<97%) bisulphite conversion efficiency, two were excluded due to missing clinical data, and five were rejected due to an inadequate amount or quality of DNA. Samples from individuals with transient islet antibodies (*n* = 47) or persistent positivity for only one islet antibody (*n* = 4) were excluded from the study (see ESM Methods for details). This resulted in a total of 122 samples for use in the analysis.

### RRBS

The library preparation steps were adapted from the RRBS protocol described by Boyle et al [7]. An Illumina HiSeq 2500 instrument (San Diego, CA, USA) was used for paired-end sequencing (2 × 100 bp) of the DNA libraries. We applied the data analysis workflow that has been described previously in more detail [8]. Briefly, a generalised mixed-effects model implemented in the R package PQLseq [9] was fitted separately for read counts at each high-coverage CpG site on autosomal chromosomes. A coverage of 10 was the minimum required in at least one third of the samples in both groups, but the median coverage was 28. The overall coverage and proportion of missing values are shown in ESM Figs 2 and 3. The covariates listed in Table 1 were modelled as fixed effects, and the genetic similarity between individuals was modelled as a random effect. The reasons for inclusion/exclusion of covariates are given in ESM Table 1. The Wald test *p* values computed within PQLseq were spatially adjusted using a weighted *Z* test implemented in the RADMeth package [10]. As the spatially adjusted *p* values were found to be inflated, the false discovery rate (FDR) was estimated empirically through a permutation analysis [8] (see ESM Methods for details).

### Alternative differential methylation analysis with a reduced number of covariates

The analysis was repeated with a reduced number of covariates such that only necessary covariates were included in the generalised mixed-effects model. These were class (case/control), HLA risk class, sex, and PC1 and PC2 (see ESM Methods for details).

### Targeted bisulphite pyrosequencing

Targets for technical validation by pyrosequencing were selected based on statistical significance in the RRBS analysis. Regions that were differentially methylated according to the DAISY study [4] and showed the same direction of difference in this study were also selected. The genomic regions of interest were amplified by 45 rounds of PCR. Bisulphite pyrosequencing was performed using the PyroMark Q24 system (Qiagen, Hilden, Germany) on 58 samples that were a subset of the samples studied with RRBS. A linear regression analysis (ANOVA) was performed for arcsin-transformed DNA methylation proportions. The explanatory variables were the same as those included in the RRBS data analysis (see ESM Methods for details).

### Ethical aspects

All participating families gave an informed consent for the genetic HLA screening from umbilical cord blood and for the follow-up. The study was originally approved by the Ethics Committee of the Hospital District of Southwest Finland, followed by the Ethics Committee of the Hospital District of Northern Ostrobothnia. The study followed the principals of the Helsinki II declaration.

## Results

Altogether, 2,568,146 CpG sites fulfilled the quality and coverage criteria and were included in the differential methylation analysis. These CpG sites covered 23,174 unique enhancer regions out of 52,563 double-elite human enhancers in the GeneHancer database, and included genomic regions that were within 2 kb of 57 risk loci for type 1 diabetes [11, 12].

**Fig. 1:**
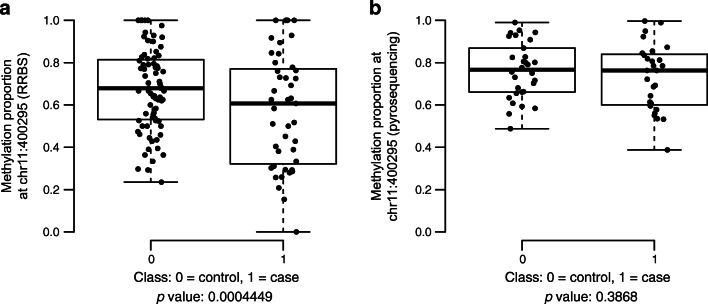
Methylation proportions at Chr11:400295 quantified using two different technologies (RRBS and targeted pyrosequencing), visualised as boxplots. (**a**) A CpG site at Chr11:400295 on an intron of *PKP3* showed weak evidence of differential methylation between case and control participants (not as an individual cytosine but as part of a candidate differentially methylated region), as measured by RRBS. (**b**) Validation by targeted pyrosequencing showed that the difference was not significant. The *p* values shown below each plot are nominal (neither spatially adjusted nor multiple testing-corrected). The midline of each boxplot is drawn at the median, boxes range from the 1st to the 3rd quartile, and whiskers extend to the most extreme values

None of the covered CpG sites were differentially methylated between the case and control participants as individual CpG sites (Benjamini–Hochberg-corrected *p* value <0.05 before spatial adjustment). After spatial adjustment, two adjacent CpG sites (chr11:400288 and chr11:400295, GRCh37 genome assembly) on an intron of gene Plakophilin-3 (*PKP3*) showed weak evidence of hypomethylation in the case participants (empirically estimated FDR <0.05, ESM Table 2). However, technical replication by targeted pyrosequencing showed that the difference was not significant (Fig. 1 and ESM Fig. 4).

The strong inflation of spatially adjusted *p* values was an important observation in these data, as described previously [8]. Before the inflation was discovered, 28 genomic regions were considered differentially methylated between cases and controls based on Benjamini–Hochberg-corrected spatially adjusted *p* values (<0.05). We carried out pyrosequencing to validate five selected targets technically, but the results did not indicate differential methylation between the groups (ESM Table 3). Empirical FDR control of the RRBS results further confirmed that the differences were indeed not significant.

In the DAISY study, observations in umbilical cord blood validated the direction of difference at genomic regions that were differentially methylated at later time points [4]. Our results did not validate the results of the DAISY study (ESM Table 4). However, methylation differences at these candidate regions were highly concordant between RRBS and pyrosequencing (ESM Table 4). Furthermore, successful technical validation of a sex-associated region confirmed that concordant results could be obtained by these two technologies (ESM Fig. 5) [8].

## Discussion

Distinct DNA methylation patterns have recently been observed to precede type 1 diabetes in whole blood collected from very young children [4]. We tested the possible presence of such differences at the time of birth in a collection of umbilical cord blood samples. Compared with previous studies, our data covered a substantially larger number of CpG sites. Based on our results, differences between children who progress to type 1 diabetes and those who remain healthy throughout childhood are not yet present in the perinatal DNA methylome. However, we cannot exclude the possibility that such differences could be found in a larger dataset. The coverage of RRBS and the statistical power to detect small (e.g. 1%) differences with these sample numbers (see ESM Methods for details) are limited to relatively CpG-rich regions.

This study was limited to an overall comparison between healthy controls and a heterogeneous group of case participants with different first-appearing autoantibodies, ages at seroconversion (range 0.5–11.6 years) and ages at diagnosis (range 1.6–18.8 years), who may represent different disease subtypes, the existence of which has been suggested by several studies during the past decade [13]. For example, the group of children with IAA as the first-appearing islet autoantibody is characterised by a different HLA DR/DQ profile and age at seroconversion compared with children with GADA as the first-appearing autoantibody [5]. Determining the epigenetic profile of newborn infants representing a potential disease subtype, for example children who develop type 1 diabetes at very young age, would be an interesting goal for future studies.

## Supplementary information

ESM 1 (PDF 1.01 mb)

## Data Availability

The datasets generated and analysed during the current study are available in the ArrayExpress repository, accession code E-MTAB-10530 (www.ebi.ac.uk/arrayexpress/experiments/E-MTAB-10530/).

## Abbreviations

DAISY: Diabetes Autoimmunity Study in the Young
DIPP: Diabetes Prediction and Prevention Study
FDR: False discovery rate
GADA: Glutamic acid decarboxylase antibody
IA-2A: Insulinoma-associated protein 2 antibody
IAA: Insulin autoantibody
RRBS: Reduced representation bisulphite sequencing
