# Supplementary material for: Umbilical cord blood DNA methylation in children who later develop type 1 diabetes
Source: Diabetologia. 2022 Jun 18;65(9):1534–40. doi: 10.1007/s00125-022-05726-1 (PMC9345803; doi:10.1007/s00125-022-05726-1)
Supplement: Supplementary file 1 — (PDF 1.01 mb) [file 125_2022_5726_MOESM1_ESM.pdf]

Electronic supplementary material (ESM):

ESM Methods and Results....................2

Power analysis....................2

Sample collection and HLA risk class determination....................3

Islet autoantibody measurement....................4

Sample inclusion criteria....................4

Reduced representation bisulfite sequencing....................4

Alternative differential methylation analysis with a reduced number of covariates....................6

Targeted bisulfite pyrosequencing....................6

References....................7

ESM Tables and Figures....................10

ESM Methods and Results

Power analysis

The power analysis was performed in simulated bisulfite sequencing data through a tool developed by Lea et al. [1]. The following parameter values were applied:

Type of predictor: Binary
Number of samples: 122
Number of samples in condition 1: 43 (the number of cases)
Type of model: Linear mixed effects model (LMM)
The proportion of variation explained by the data structure (not by the variable of interest): 0.5
Coverage range: 10 – 50
Proportion of variation explained by the variable of interest (case/control): 0.25
False discovery rate (FDR) threshold: 0.05
Proportion of the dataset simulated as true positives: 0.1

The relatedness matrix (relatedness between individuals based on correlations between their SNP profiles) was utilized as the data structure matrix. To be conservative, we assumed the data structure would explain a much larger proportion of the variation than the variable of interest (case/control) at sites that were simulated as true positives. Based on this simulation, the power to detect true positives was estimated to be 89.6 % (ESM Fig. 1) at FDR 0.05. Compared to this simulation, statistical power in the actual analysis was increased by adjusting for the spatial correlation between CpG sites. However, small methylation differences (e.g. 1 %) at individual CpG sites (that do not belong to a differentially methylated region) are not likely to be discovered in this study.

The power to detect differential methylation in real data with our workflow has been demonstrated in our preprint on sex-associated DNA methylation [2]. However, since the sample number was slightly lower in the present study, we re-evaluated the statistical power by repeating the differential methylation analysis between the sexes in these 122 samples (42 females vs. 80 males, while accounting for all the covariates listed in the main text Table 1) and comparing the results to two independent published studies on sex-associated umbilical cord blood DNA methylation that were based on a different technology (methylation microarrays) and different populations [3,4]. With 122 samples we were able to detect 3935 sex-associated CpG sites (FDR < 0.05) out of which 247 were covered by Illumina 450K methylation microarrays (and hence could have been detected by the earlier studies). We were able to confirm 145 sex-associated CpG sites out of 1675 CpG sites that were associated with sex in one or both earlier studies [3,4] and were covered by our RRBS data. The overlap was highly significant (Fisher's exact test p value $< 2.2 \times 10^{-16}$). These 145 confirmed sex-associated CpG sites included sites with very small effect sizes. Their absolute coverage-corrected mean methylation differences between the sexes ranged from 0.4 % to 20.4 %, median 9.4 %.

Sample collection and HLA risk class determination

Umbilical cord blood was collected immediately after birth in 3 ml K3-EDTA tubes in the delivery room at the Turku University Hospital and transferred to the DIPP study centre where it was stored at -20°C. After receiving informed consent from the parents, each sample was transferred to the immunogenetics laboratory (destroyed in case informed consent was not received), where it was thawed, and a drop of blood was transferred to a sample collection card for HLA genotype determination. The remaining sample was stored at -20°C. DNA was extracted by applying salting out procedure [5]. HLA-DR-DQ genotypes were determined from the DNA on the dried blood spots using assays that were designed to densely probe the genomic regions associated with type 1 diabetes. The genotyping was started from major DQB1 alleles. A hypervariable region on the second exon was further sequenced for individuals with certain DQB1 haplotypes. A detailed description of the genotyping procedure and the risk class definitions have been reported earlier [6,7]. The genotypes corresponding to each risk class within this study are:

High (any of the following):
(DR3) - DQA1*05 - DQB1*02 / DRB1*0401 - DQA1*03 - DQB1*0302
(DR3) - DQA1*05 - DQB1*02 / DRB1*0404 - DQA1*03 - DQB1*0302

Moderate (any of the following):
(DR3) - DQA1*05 - DQB1*02 / (DR3) - DQA1*05 - DQB1*02
(DR7) - DQA1*0201 - DQB1*02 / DRB1*0401 - DQA1*03 - DQB1*0302
DRB1*0401 - DQA1*03 - DQB1*0302 / (DR1/10) - DQB1*0501
DRB1*0401 - DQA1*03 - DQB1*0302 / (DR13) - DQB1*0604
DRB1*0401 - DQA1*03 - DQB1*0302 / (DR13) - DQB1*0609
DRB1*0401 - DQA1*03 - DQB1*0302 / (DR16) - DQB1*0502
DRB1*0401 - DQA1*03 - DQB1*0302 / (DR8) - DQB1*04
DRB1*0401 - DQA1*03 - DQB1*0302 / (DR9) - DQA1*03 - DQB1*030
DRB1*0401 - DQA1*03 - DQB1*0302 / DRB1*0404 - DQA1*03 - DQB1*0302

Slightly elevated (any of the following):
(DR3) - DQA1*05 - DQB1*02 / (DR1/10) - DQB1*0501
(DR3) - DQA1*05 - DQB1*02 / (DR13) - DQB1*0604
(DR3) - DQA1*05 - DQB1*02 / (DR8) - DQB1*04
(DR3) - DQA1*05 - DQB1*02 / (DR9) - DQA1*03 - DQB1*0303
(DR7) - DQA1*0201 - DQB1*02 / DRB1*0404 - DQA1*03 - DQB1*0302
DRB1*0401 - DQA1*03 - DQB1*0302 / DRB1*0403 - DQA1*03 - DQB1*0302
DRB1*0404 - DQA1*03 - DQB1*0302 / (DR1/10) - DQB1*0501
DRB1*0404 - DQA1*03 - DQB1*0302 / (DR8) - DQB1*04
DRB1*0404 - DQA1*03 - DQB1*0302 / (DR9) - DQA1*03 - DQB1*0303

Neutral (any of the following):
(DR1/10) - DQB1*0501 / (DR1/10) - DQB1*0501
(DR3) - DQA1*05 - DQB1*02 / DRB1*0403 - DQA1*03 - DQB1*0302
DRB1*0401 - DQA1*03 - DQB1*0302 / (DR14) - DQB1*0503

DRB1*0401 - DQA1*03 - DQB1*0302 / (DR7) - DQA1*0201 - DQB1*0303
DRB1*0404 - DQA1*03 - DQB1*0302 / (DR13) - DQB1*0603
DRB1*0405 - DQA1*03 - DQB1*0302 / (DR7) - DQA1*0201 - DQB1*0303

Study participants were invited to the follow-up based on an increased risk to develop type 1 diabetes, according to the HLA risk class definitions at their time of birth. HLA risk class definitions have been updated during the study years to increase the sensitivity and specificity of the screening [6,7].

Islet autoantibody measurement

During DIPP follow-up, serum samples for islet autoantibody measurements were collected at 3-month intervals until the age of 2 years and thereafter every 6 or 12 months until the age of 15 years or until type 1 diabetes diagnosis. Islet autoantibodies were measured with specific radio-binding assays and included IAA (insulin autoantibody), IA-2A (insulinoma-associated protein 2 antibody), GADA (glutamic acid decarboxylase antibody) and ZnT8A (zinc transporter-8 antibody). Classical islet cell antibodies (ICA) were used as the only autoantibody screening method for DIPP children born until the end 2002, and if positive, all other autoantibodies were measured from all earlier and future samples of the child. ICA, IAA, GADA and IA-2A were measured from all follow-up samples from children born since 2003, whereas ZnT8A were measured only if at least one of the other autoantibodies became positive [8]. In addition, all five islet autoantibodies were measured from all samples of the first 1000 children participating in DIPP follow-up [8].

Sample inclusion criteria

Out of 200 umbilical cord blood DNA samples within this analysis, 20 were rejected due to low (< 97 %) bisulfite conversion efficiency, two were excluded due to missing clinical data, and five were rejected due to inadequate amount or quality of DNA. Samples from individuals with any transient islet antibodies (N = 47) or persistent positivity for only one islet antibody (N = 4) were excluded from this study on type 1 diabetes but included in our study on perinatal DNA methylation associated with other variables [2].

Reduced representation bisulfite sequencing

DNA concentrations were measured with Thermo Scientific NanoDrop 2000, and the samples were purified with Genomic DNA Clean & Concentrator -10/ ZR-96 Genomic DNA Clean & Concentrator -5 plate kits (Zymo Research, cat. nos D4010 and D4066) according to the protocols for each kit. Library preparation was started from 200 ng of genomic DNA, and *E. coli* genomic DNA (USB, cat. no. 14380) was used as a carrier, 50 ng/library. Library preparation protocol for reduced representation bisulfite sequencing (RRBS) was adapted from [9]. As suggested by the protocol [9], a lower concentration of adapters (1:10 dilution) was used than recommended by the manufacturer to reduce the occurrence of adapter dimers. Bisulfite conversion and sample purification were carried out according to the Invitrogen MethylCode Bisulfite Conversion Kit protocol. Aliquots of converted DNA were amplified by 18 cycles of PCR with Taq/Pfu Turbo Cx Polymerase, a proofreading PCR enzyme that does not stall at

uracil. PCR-amplified RRBS libraries were extracted using two subsequent rounds of SPRI bead clean-ups to minimize primer dimers in the final libraries. The high quality of the libraries was confirmed with either Advanced Analytical Fragment Analyzer or Bioanalyzer, depending on the size of the prepared library batch. The concentrations of the libraries were quantified with Qubit® Fluorometric Quantitation, Life Technologies, and only high-quality libraries were sequenced.

The samples were normalized and pooled for the automated cluster preparation which was carried out with Illumina cBot station. The libraries were run in 32 lanes, four-seven samples per lane. The samples were sequenced with Illumina HiSeq 2500 instrument using TruSeq v3 sequencing chemistry. Paired-end sequencing with 2 x 100 bp read length was used with 6 bp index run. The technical quality of the HiSeq 2500 run was high and the cluster amount was as expected. A minimum of 85 % of all bases above Q30 was required. The yields were 18 - 37 million raw paired-end reads per sample. The base calling was performed using Illumina's bcl2fastq2 software, the output of which is of standard fastq format.

The code for the data analysis is available in GitHub [10]. TrimGalore [11] and Bismark [12] were used for trimming and alignment of paired-end RRBS reads on the GRCh37 (hg19) genome assembly [13]. After removing M-biases, potential single nucleotide polymorphisms [14], and sites with extremely high coverages, a minimum coverage of 10 reads was required for at least one third of the samples in both groups. The coverage distribution and a distribution of missing values per CpG site in the filtered data are presented in ESM Fig. 2 and 3.

To evaluate the coverage of potential genomic regions of interest, we downloaded all high-confidence enhancers that had evidence for interactions with genes from multiple sources ("double elite regulatory elements") for human chromosomes 1 – 22 from the GeneHancer database [15] through UCSC table browser, GRCh37 (hg19) genome assembly, accessed on January 25th 2022 [16]. R package GenomicRanges [17] was utilized to investigate the overlap between these enhancers and our high-coverage CpG sites (2568146 CpG sites that fulfilled the above-mentioned criteria). We also computed the distances between the high-coverage CpG sites and type 1 diabetes risk loci [18], excluding loci in the HLA region, through GenomicRanges.

For the differential methylation analysis between the cases and controls, a generalized mixed effects model (GLMM) implemented in the R package PQLseq [19] was fit separately for read counts at each CpG site on autosomal chromosomes. The covariates listed in the main text Table 1 were modeled as fixed effects and the genetic similarity between individuals as a random effect. Since we could not know, which covariates might be important confounding factors, we included all available reliably recorded information. However, only one covariate was selected from each group of mutually correlated clinical covariates (detailed inclusion criteria are listed in ESM Table 1). We also repeated the analysis with a reduced number of covariates, as described below. A few missing covariate values were median-imputed, and continuous covariates were Z-transformed. The Wald test p values computed within PQLseq were spatially adjusted by a weighted Z-test implemented in package RADMeth [20]. Since the spatially adjusted p values

were found to be inflated, false discovery rate (FDR) was estimated empirically through a permutation analysis [2].

Alternative differential methylation analysis with a reduced number of covariates

The differential methylation analysis was repeated such that only necessary covariates were included in the GLMM. These were:

Class (case/control): The variable of interest

HLA risk class: This was the only clinical variable that correlated with class. We included it as a confounding covariate because the goal was to study DNA methylation associated with type 1 diabetes, independent of HLA risk that has already been thoroughly studied.

Sex: Sex was not significantly unbalanced between the groups (apart from HLA risk, neither was any other variable with available data – not even nominally). However, it was the only clinical variable that was clearly associated with differential methylation in these data, as described earlier [2]. It was therefore included in the reduced model to make sure the results would not be confounded by the slight unbalance between groups (40 % female in the case group vs. 32 % female in the control group).

PC 1 and PC 2: the projection of the data on the first two principal components was included to account for technical variation and variation in cell type composition. In these data, PC 1 and PC 2 were observed to correlate with library preparation batches and bisulfite conversion efficiency but not with any clinical variable.

The top two CpG sites with smallest spatially adjusted P-values were the same as those from the above-described analysis with the full model (that included all covariates listed in main text Table 1): chr11:400288 and chr11:400295 (GRCh37 genome assembly) – the only CpG sites that showed even weak evidence of differential methylation as part of a candidate differentially methylated region, based on spatially adjusted *p* values with an empirically determined threshold at 5.33×10^{-13} (ESM Fig. 4, ESM Table 2).

The results were concordant between the simple and the full model. Both models agreed that none of the CpG sites showed evidence of differential methylation based on Benjamini-Hochberg corrected *p* values (before the spatial adjustment). The correlation between $\log_{10} p$ values was 0.80, and the overlap of top 10 CpG sites ranked by *p* value was 3. The correlation between spatially adjusted *p* values was also 0.80, and the overlap of top 10 CpG sites ranked by spatially adjusted *p* value was 7.

Targeted bisulfite pyrosequencing

Targets for technical validation by bisulfite pyrosequencing were selected based on statistical significance in the RRBS analysis. Also, regions that were differentially methylated according to the DAISY study [21] and showed the same direction of difference in this study, were selected.

For this analysis, 28 case subjects and 30 control subjects were included, based on the following selection criteria: Amount of remaining DNA (determined by Qubit™ dsDNA HS Assay Kit, Invitrogen Ref Q32854), full-term (gestational age ≥ 37 weeks), similar sex distributions in both groups, normal birth weight (2.5 – 4.5 kg), no multiple pregnancies, normal Apgar points (8 – 10), no perinatal asphyxia, vaginal birth and no maternal smoking. PyroMark assay design 2.0 software (Qiagen) was used to design the assays for the methylation validation for the selected targets. Target specific primers designed with the software include primer pair with site-specific biotinylation for target amplification and pyrosequencing primer. The regions of interest were those listed in ESM Tables 2-4, and region chr17:38024237–chr17:38024290 (hg19 coordinates) which included six CpG sites that were differentially methylated between sexes. The sex-associated target was selected as a positive control to confirm that concordant results could be obtained with two technologies (RRBS and targeted pyrosequencing). The sequencing was done on three to five batches such that a roughly even numbers of male and female case and control subjects were allocated to each batch.

The samples were prepared for pyrosequencing from 200 ng of DNA per sample. Bisulfite treatment was performed using EZ DNA Methylation-GoldTM Kit (Zymo Research cat no D5006) following the manufacturer's instructions. The targets were then amplified using PyroMark PCR Kit (Qiagen cat no 978703). The following PCR conditions were used for amplifications: initial denaturation at 95 °C for 15 min, 45 cycles at 94 °C for 30 s, annealing at 56 °C for 30 s, extension at 72 °C for 30 s and final extension at 72 °C for 10 min. For pyrosequencing reaction, biotin-labeled template strand was used with specific primers. Pyrosequencing was performed with PyroMark Q24 system (Qiagen) with PyroMark Q24® Advanced CpG Reagents (Qiagen cat no 970922). Methylation percentages were extracted using PyroMark Q24 Advanced 3.0.1 software.

A linear regression model was fit for each DNA methylation proportion, after applying a transformation: arcsin(2 × proportion – 1). The explanatory variables were the same as those included in the RRBS data analysis. Each model was fit with and without the covariate of interest, and the significance of the association was estimated based on an ANOVA test comparing the two models.

References

1. Lea AJ, Vilgalys TP, Durst PAP, Tung J. Maximizing ecological and evolutionary insight in bisulfite sequencing data sets. Nat Ecol Evol. 2017;1: 1074–1083.

2. Laajala E, Halla-aho V, Grönroos T et al. Permutation-based significance analysis reduces the type 1 error rate in bisulfite sequencing data analysis of human umbilical cord blood samples. Epigenetics. 2022 https://doi.org/10.1080/15592294.2022.2044127

3. Yousefi P, Huen K, Davé V, Barcellos L, Eskenazi B, Holland N. Sex differences in DNA methylation assessed by 450 K BeadChip in newborns. BMC Genomics. 2015;16: 911.

4. Maschietto M, Bastos LC, Tahira AC, Bastos EP, Euclydes VLV, Brentani A, et al. Sex differences in DNA methylation of the cord blood are related to sex-bias psychiatric diseases. Sci Rep. 2017;7: 44547.

5. Miller SA, Dykes DD, Polesky HF. A simple salting out procedure for extracting DNA from human nucleated cells. Nucleic Acids Res. 1988;16: 1215.

6. Ilonen J, Kiviniemi M, Lempainen J, Simell O, Toppari J, Veijola R, et al. Genetic susceptibility to type 1 diabetes in childhood - estimation of HLA class II associated disease risk and class II effect in various phases of islet autoimmunity. Pediatric Diabetes. 2016. pp. 8–16. doi:10.1111/pedi.12327

7. Mikk M-L, Kiviniemi M, Laine A-P, Härkönen T, Veijola R, Simell O, et al. The HLA-B*39 allele increases type 1 diabetes risk conferred by HLA-DRB1*04:04-DQB1*03:02 and HLA-DRB1*08-DQB1*04 class II haplotypes. Hum Immunol. 2014;75: 65–70.

8. Pöllänen PM, Ryhänen SJ, Toppari J, Ilonen J, Vähäsalo P, Veijola R, et al. Dynamics of islet autoantibodies during prospective follow-up from birth to age 15 years. J Clin Endocrinol Metab. 2020;105: e4638–e4651.

9. Boyle P, Clement K, Gu H, Smith ZD, Ziller M, Fostel JL, et al. Gel-free multiplexed reduced representation bisulfite sequencing for large-scale DNA methylation profiling. Genome Biol. 2012;13: R92.

10. Laajala E. In: RRBS workflow [Internet]. 11 May 2021 [cited 11 May 2021]. Available: https://github.com/EssiLaajala/RRBS_workflow

11. Krueger F. TrimGalore. A wrapper around Cutadapt and FastQC to consistently apply adapter and quality trimming to FastQ files, with extra functionality for RRBS data. TrimGalore (accessed on 27 August 2019). 2016.

12. Krueger F, Andrews SR. Bismark: a flexible aligner and methylation caller for Bisulfite-Seq applications. Bioinformatics. 2011;27: 1571–1572.

13. Church DM, Schneider VA, Graves T, Auger K, Cunningham F, Bouk N, et al. Modernizing reference genome assemblies. PLoS Biol. 2011;9: e1001091.

14. Gao S, Zou D, Mao L, Liu H, Song P, Chen Y, et al. BS-SNPer: SNP calling in bisulfite-seq data. Bioinformatics. 2015;31: 4006–4008.

15. Fishilevich S, Nudel R, Rappaport N, Hadar R, Plaschkes I, Iny Stein T, et al. GeneHancer: genome-wide integration of enhancers and target genes in GeneCards. Database (Oxford). 2017;2017. doi:10.1093/database/bax028

16. Karolchik D, Hinrichs AS, Furey TS, Roskin KM, Sugnet CW, Haussler D, et al. The UCSC Table Browser data retrieval tool. Nucleic Acids Res. 2004;32: D493-6.

17. Lawrence M, Huber W, Pagès H, Aboyoun P, Carlson M, Gentleman R, et al. Software for computing and annotating genomic ranges. PLoS Comput Biol. 2013;9: e1003118.

18. Chiou J, Geusz RJ, Okino M-L, Han JY, Miller M, Melton R, et al. Interpreting type 1 diabetes risk with genetics and single-cell epigenomics. Nature. 2021;594: 398–402.

19. Sun S, Zhu J, Mozaffari S, Ober C, Chen M, Zhou X. Heritability estimation and differential analysis of count data with generalized linear mixed models in genomic sequencing studies. Bioinformatics. 2019;35: 487–496.

20. Dolzhenko E, Smith AD. Using beta-binomial regression for high-precision differential methylation analysis in multifactor whole-genome bisulfite sequencing experiments. BMC Bioinformatics. 2014;15: 215.

21. Johnson RK, Vanderlinden LA, Dong F, Carry PM, Seifert J, Waugh K, et al. Longitudinal DNA methylation differences precede type 1 diabetes. Sci Rep. 2020;10: 3721.

ESM Tables and Figures

ESM Table 1, modified from [2]**: Description of variables with available data.** Column 2 indicates whether the variable was included in the differential methylation analysis, which was done with a generalized linear mixed-effects model (GLMM). One clinical covariate from each group of mutually correlating covariates was included in the differential methylation analysis to account for potential confounding effects. Pearson correlations greater than 0.3 (in absolute value) with p value < 0.05 or alternatively Fisher's exact test p value < 0.05 (for pairs of binary covariates) were considered relevant. We prioritized continuous covariates and binary covariates with sufficient sample numbers in both groups. We avoided including covariates that contained missing values or are difficult to measure, such as the duration of delivery phase 1.

**Covariate(s)**	**Included in regression (yes/no)**	**Details and reasons for inclusion/exclusion**
Age, mother	Yes	The maternal age correlates only with the number of earlier pregnancies and deliveries, and is prioritized over them, since continuous covariates are easier to model than counts.
Birth length	No	Correlates with birth weight
Birth weight	Yes	Birth weight is included to represent the child's size. It is prioritized over birth height, head circumference and pregnancy duration, since birth weight has the best measurement accuracy.
BMI, mother	Yes	This is the pre-pregnancy body mass index.
Class (case/control)	Yes	The goal was to study differential methylation associated with this covariate. Case subjects were diagnosed with type 1 diabetes or became persistently positive for at least two islet autoantibodies, whereas control individuals remained completely negative for islet autoantibodies throughout the follow-up.
Caesarean section	Yes	The mode of delivery was simplified to vaginal/C-section. C-section only correlates with perinatal asphyxia and is prioritized, since it is simple to define and does not include any measurement uncertainty
Age at diagnosis, age at seroconversion, first-appearing autoantibodies	No	Only relevant for case individuals
Duration of delivery stage 1	No	Difficult to measure and includes too many missing values (22 out of 122), for example for all C-section cases. Also correlates with the usage of epidural anesthetic
Duration of delivery stage 2	No	Too many missing values (19 out of 122), for example at all C-section cases. Also correlates with the usage of epidural anesthetic
Gestational vaginal bleeding	No	A binary variable, correlates with induced labor

Gestational weight gain, mother	Yes	Does not correlate with any other variable with available data
Glucose tolerance test result, mother	No	A binary variable, correlates with insulin-treated diabetes and includes many missing values (101 out of 122)
Head circumference	No	Correlates with birth weight
Height, mother	Yes	Included to represent the maternal size together with the BMI
HLA risk class	Yes	Since the goal was to study differential methylation associated with later disease progression, independent of the well-studied HLA risk, HLA risk was included as a confounding covariate. The original data included four levels (neutral, slightly elevated, moderate, high) but neutral and slightly elevated were merged into one category. In practice, “HLA risk neutral” and “HLA risk high” were modeled as separate binary covariates (moderate was included in the intercept)
Induced labor	Yes	Correlates only with gestational vaginal bleeding and is prioritized, since induced labor is simple to define, whereas gestational vaginal bleeding can have different degrees of severity
Infant weight when discharged	No	Correlates with birth weight and is not easily comparable between different individuals, since they spend variable amounts of time in the hospital
Insulin-treated diabetes, mother	Yes	This can be insulin-treated diabetes of any type (for example gestational diabetes). This covariate is prioritized over neonatal intensive care, neonatal hypoglycemia, and earlier C-section, since insulin-treatment throughout the pregnancy is expected to be more relevant for umbilical cord blood than events that take place before or after the pregnancy.
Library preparation batch	Yes	This is a categorical covariate with seven levels (transformed to six binary covariates + intercept). A median number of 16 samples (range 4 – 32) were processed within the same batch. The samples were allocated to the batches, such that each batch contained a comparable group of cases and controls.
Low Apgar points	Yes	The 1-minute Apgar points were used here as a binary variable (0=normal, 1=low). Values 7 and lower were considered low
Multiple pregnancy	No	This binary variable correlates with birth weight, and the data only includes a few multiple pregnancies.
Neonatal hypoglycemia	No	Correlates with maternal insulin-treated diabetes

Neonatal intensive care	No	Correlates with maternal insulin-treated diabetes
Number of earlier C-sections	No	The number of earlier Caesarean sections was simplified to a binary variable (0=none, 1=one or more). It correlates with maternal insulin-treated diabetes.
Number of earlier deliveries	No	The number of earlier deliveries was simplified to a binary variable (0=none, 1=one or more). It correlates with the maternal age.
Number of earlier miscarriages	Yes	The number of earlier miscarriages was simplified to a binary variable (0=none, 1=one or more). There was no reason to exclude this covariate, since it only correlated with the number of earlier pregnancies.
Number of earlier pregnancies	No	The number of earlier pregnancies was simplified to a binary variable (0=none, 1=one or more). It correlates with the maternal age.
Perinatal asphyxia	No	Correlates with C-section
Pregnancy duration	No	Correlates with birth weight
Principal components 1 and 2	Yes	The principal components analysis (PCA) on the coverage-filtered methylation proportion matrix was done after median-imputing remaining missing values for each CpG site. The projection of the data on principal components 1 and 2 was included in the model to represent technical variation.
Sex	Yes	Correlates with no other covariate
Smoking during pregnancy	Yes	The data included seven mothers smoking throughout the pregnancy and one mother smoking only during the first trimester. This variable was simplified to 0=no smoking, 1=smoking. Covariates related to in-utero conditions were generally prioritized.
Transformed month	Yes	This is the month of birth, cosine-transformed ($\cos(2\pi m/12)$, where m is the month as numbers 1 – 12)
Umbilical arterial blood pH	No	Too many missing values (18 out of 122)
Usage of epidural anesthetic	Yes	This is a binary variable indicating whether epidural anesthetic was used during delivery stage 1. The value was corrected from 1 to 0 for two individuals with an elective Caesarean section. This variable was prioritized over duration of delivery stages 1 and 2 since usage of epidural anesthetic is simple to define and does not include missing values or measurement uncertainty.
Year of birth (= sample collection year)	Yes	Included to account for possible technical variation related to storage time at -20°C

ESM Table 2: Statistics on two CpG sites in the intron of *PKP3*. Two adjacent CpG sites on the intron of *PKP3* (GRCh37 genome assembly) showed weak evidence of hypomethylation in the case group (N=43), as compared to the control group (N=79). Spatially adjusted *p* value 5.33×10^{-13} was estimated to correspond to false discovery rate 0.05, based on a permutation analysis. Coverage-corrected mean methylation difference is calculated as sum(number of methylated reads in cases) / sum(number of total reads in cases) – sum(number of methylated reads in controls) / sum(number of total reads in controls). The full model included all covariates listed in the main text Table 1, whereas the simple model included only HLA risk class, sex, and PCs 1-2.

	chr11:400288	chr11:400295
P value, full model	1.14×10^{-2}	4.45×10^{-4}
Spatially adjusted *p* value, full model	1.22×10^{-13}	2.64×10^{-13}
p value, simple model with a reduced number of covariates	1.39×10^{-2}	7.27×10^{-3}
Spatially adjusted *p* value, simple model with a reduced number of covariates	2.98×10^{-13}	5.56×10^{-13}
Total number of reads, case group	1268	1109
Number of methylated reads, case group	649	615
Total number of reads, control group	2349	2173
Number of methylated reads, control group	1399	1442
Coverage-corrected mean methylation difference between groups	–0.084	–0.109

ESM Table 3: Summary statistics of genomic regions that were chosen for targeted validation by pyrosequencing based on Benjamini-Hochberg-corrected spatially adjusted *p* values before the *p* value inflation was discovered. Based on pyrosequencing, these regions showed no significant differences between the case group and the control group. The empirical FDR control later revealed that the differences were not truly significant in RRBS data either.

Description of the target(s)	Exon and intron of *GFI1*	Exon of *CHD7*	Intron of *BRSK2*	Intron of *NFATC1*
RRBS: candidate differentially methylated region	chr1:92946521-92946810	chr8:61777859-61777938	chr11:1412936-1413023	chr18:77284553-77284613
RRBS: Largest difference between cases and controls	0.169	0.175	-0.208	0.143
RRBS: Smallest raw *p* value	0.0074	0.0011	0.00033	0.0047
RRBS: Smallest spatially adjusted *p* value	2.90E-07	5.00E-06	3.70E-05	6.40E-06
Pyrosequenced region(s)	chr1:92946620-92946521 and chr1:92946641-92946752	chr8:61777937-61777909	chr11:1412927-1413022	chr18:77284550-77284612

Number of CpG sites within pyrosequenced region(s)	22	4	3	2
Pyrosequencing: largest difference between cases and controls	0.052	0.023	-0.097	0.028
Pyrosequencing: smallest raw *p* value	0.23	0.094	0.63	0.028

ESM Table 4, modified from [21]**: Comparison between our results and the results from the DAISY study.** According to the study by Johnson et al. [21], observations in umbilical cord blood confirmed the direction of difference at genomic regions that were differentially methylated at later time points, although very few of these differences were statistically significant in cord blood. We did not observe concordant direction of difference between our study and the study by Johnson et al. The three regions that showed the same direction of difference between these studies were validated by targeted bisulfite pyrosequencing. Our RRBS and pyrosequencing results were highly concordant.

Region (differentially methylated according to Johnson et al.)	Nearest gene	Avg. Beta (between cases and controls, Johnson et al.)	Pyrosequenced region	Observations from our data, NA means the region was not covered by RRBS
chr9:124989241-124990457	LHX6	0.071		Not confirmed (mean difference is to the opposite direction)
chr4:57547347-57548094	HOPX	0.032		NA
chr12:96350519-96350796	AMDHD1	-0.022		Not confirmed (methylation differences to both directions within region)
chr17:47287410-47287578	ABI3	0.012		NA
chr8:1113291-1113433	ERICH1-AS1	0.037		Not confirmed (methylation differences to both directions within region)
chr2:43903227-43903651	AC011242.6	-0.013	chr2:43903574-43903595	Direction of difference confirmed by RRBS (negative in 10 out of 12 CpGs, mean -0.008) as well

				as pyrosequencing (negative in 4 out of 4 CpGs, mean -0.007)
chr2:177014849-177015126	MIR10B	-0.013		Not confirmed (methylation differences to both directions within region)
chr12:1725788-1726077	FBXL14	-0.013		Not confirmed (methylation differences to both directions within region)
chr8:1649868-1650173	DLGAP2	0.026		NA
chr8:1012324-1012466	CTD-2281E23.2	0.013		NA
chr6:28973328-28973521	ZNF311	-0.0050		NA
chr2:202901352-202901471	FZD7	0.071	chr2:202901422-202901438	Direction of difference confirmed by RRBS (positive in 7 out of 7 CpG sites, mean 0.06), as well as pyrosequencing (positive in 3 out of 3 CpG sites, mean 0.08)
chr8:1273604-1273857	CTD-2281E23.1	0.021		NA
chr22:31002892-31003148	TCN2 and PES1	-0.0035	chr22:31002982-31002991	Direction of difference confirmed by RRBS (negative in 4 out of 5 CpG sites, mean -0.001). Differences at two pyrosequenced sites were 0.00002 and -0.0009
chr7:94953810-94954203	AC004022.7	-0.042		Not confirmed (mean difference is to the opposite direction)
chr17:1395864-1396124	MYO1C	0.017		Not confirmed (methylation differences to both directions within region)
chr2:1452260-1452368	TPO	0.018		NA

chr18:19756877-19757023	RP11-627G18.2	-0.0058		Not confirmed (methylation differences to both directions within region)
chr10:3466795-3466853	RP11-482E14.1	0.029		Not confirmed (mean difference is to the opposite direction)
chr16:1060367-1060559	RP11-161M6.3	0.016		Not confirmed (methylation differences to both directions within region)
chr19:35800589-35800744	MAG	-0.027		Not confirmed (methylation differences to both directions within region)
chr7:12443880-12444116	VWDE	-0.028		NA
chr13:111956623-111956696	ARHGEF7	0.014		NA
chr17:45924888-45925061	SP6	-0.022		Not confirmed (mean difference is to the opposite direction)
chr7:73157217-73157384	ABHD11	0.037		Not confirmed (methylation differences to both directions within region)
chr20:17296317-17296451	PCSK2	-0.011		Not confirmed (methylation differences to both directions within region)
chr17:42733527-42733699	C17orf104	-0.012		Not confirmed (mean difference is to the opposite direction)
chr16:89164953-89164991	ACSF3	-0.011		NA

ESM Fig. 1: A QQ-plot of simulated -$\log_{10}$ *p* values, compared to a uniform distribution. This is a visualization of statistical power, based on simulated bisulfite sequencing data, generated by the tool by Lea et al. [1]. The simulated data was generated with coverage range 10 – 50 with 43 and 79 samples in each group, corresponding to our numbers of cases and controls. The proportion of variation explained by the group (case/control) was assumed to be 0.25, while the proportion of variation explained by other data structure was assumed to be 0.5. The proportion of data simulated as true positives was 0.1. The differential methylation analysis was performed with a linear mixed effects model with FDR threshold 0.05. The power to detect true positives was estimated to be 89.6 %. The amount of detected negatives (falsely detected as positives) was 0.756 %. The average difference between the groups was 18.4 % in this simulation.

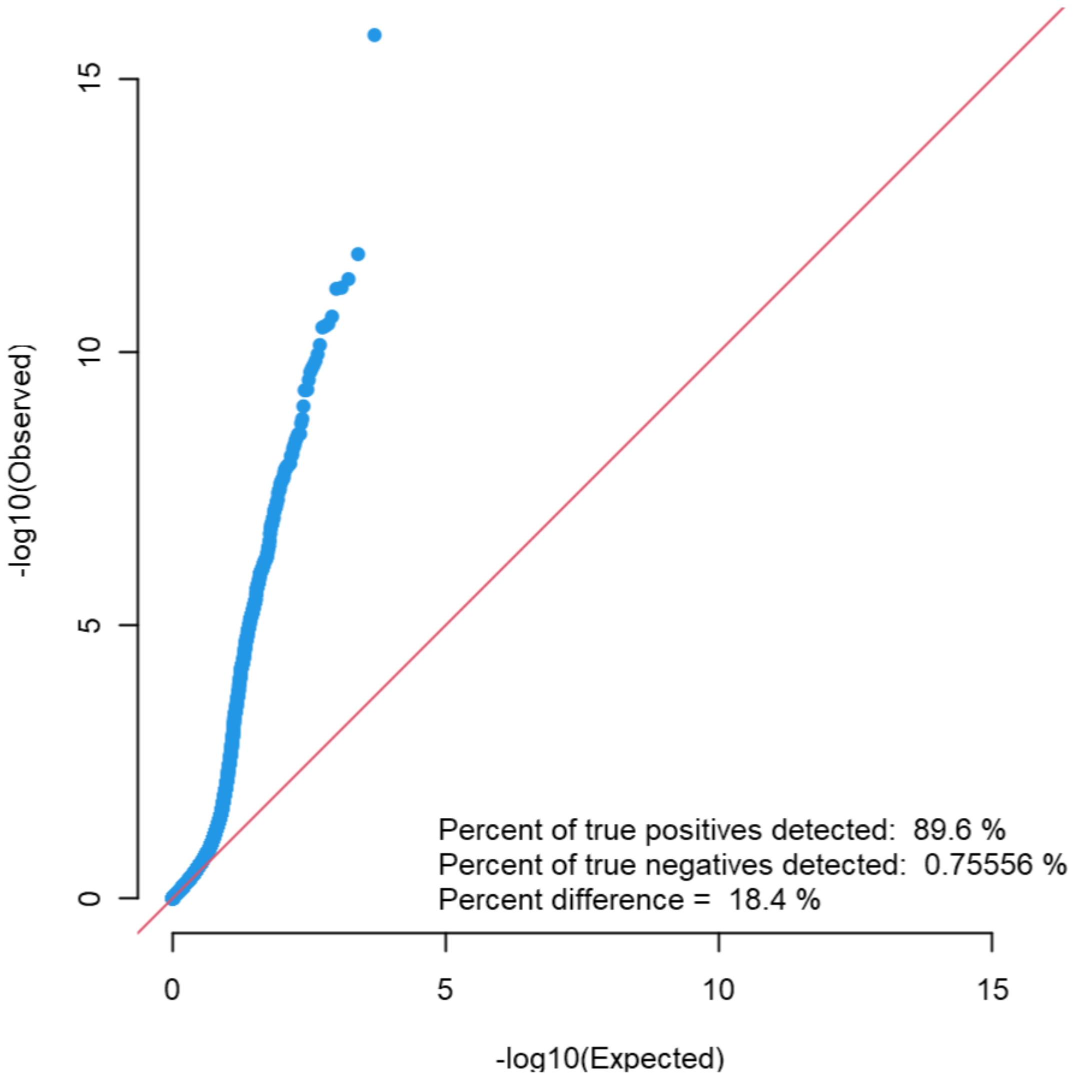

ESM Figure 2: A histogram of coverage (total number reads) across all measurements (2568146 CpG sites × 122 samples) that were part of the differential methylation analysis. The median coverage was 28.

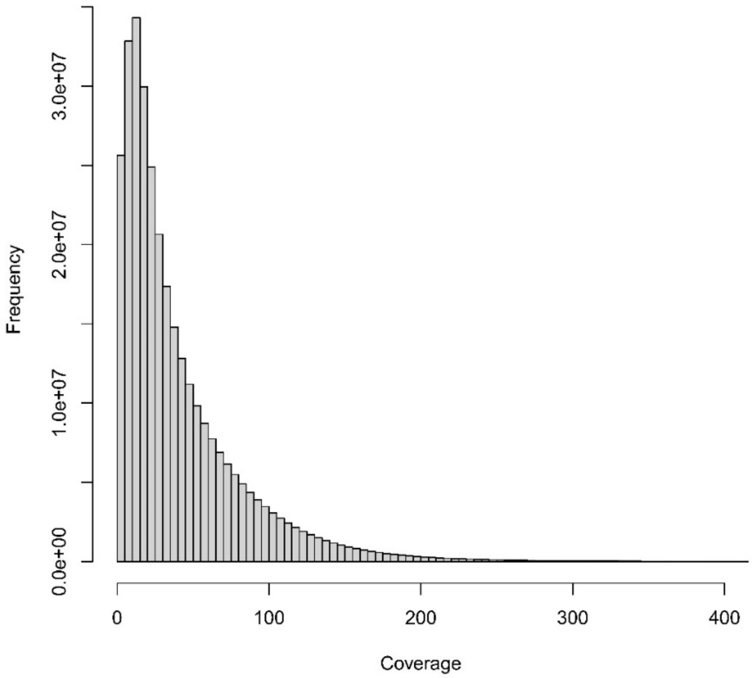

ESM Figure 3: A histogram of the number of missing (coverage = 0) values across 2568146 CpG sites that were part of the differential methylation analysis. The median number of missing values was 0.

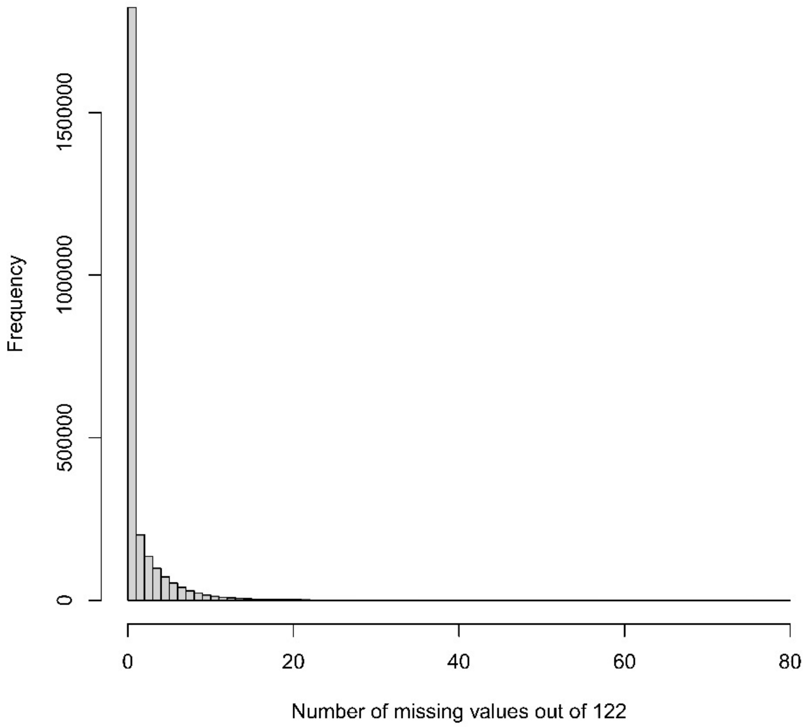

ESM Figure 4: Two CpG sites on the intron of Plakophilin 3 (*PKP3*) showed weak evidence of differential methylation between cases and controls (not as individual CpG sites but as part of a region with spatially adjusted *p* values in the order of 10^{-13}). However, technical replication by pyrosequencing revealed that the differences were not significant. The RRBS and pyrosequencing results are presented here as boxplots. The raw *p* values for differential methylation (based on GLMM for RRBS and ordinary linear model for pyrosequencing, separately at each CpG site) are marked below each plot. The midline of each boxplot is drawn at the median, boxes range from the 1st to the 3rd quartile, and whiskers extend to the most extreme values.

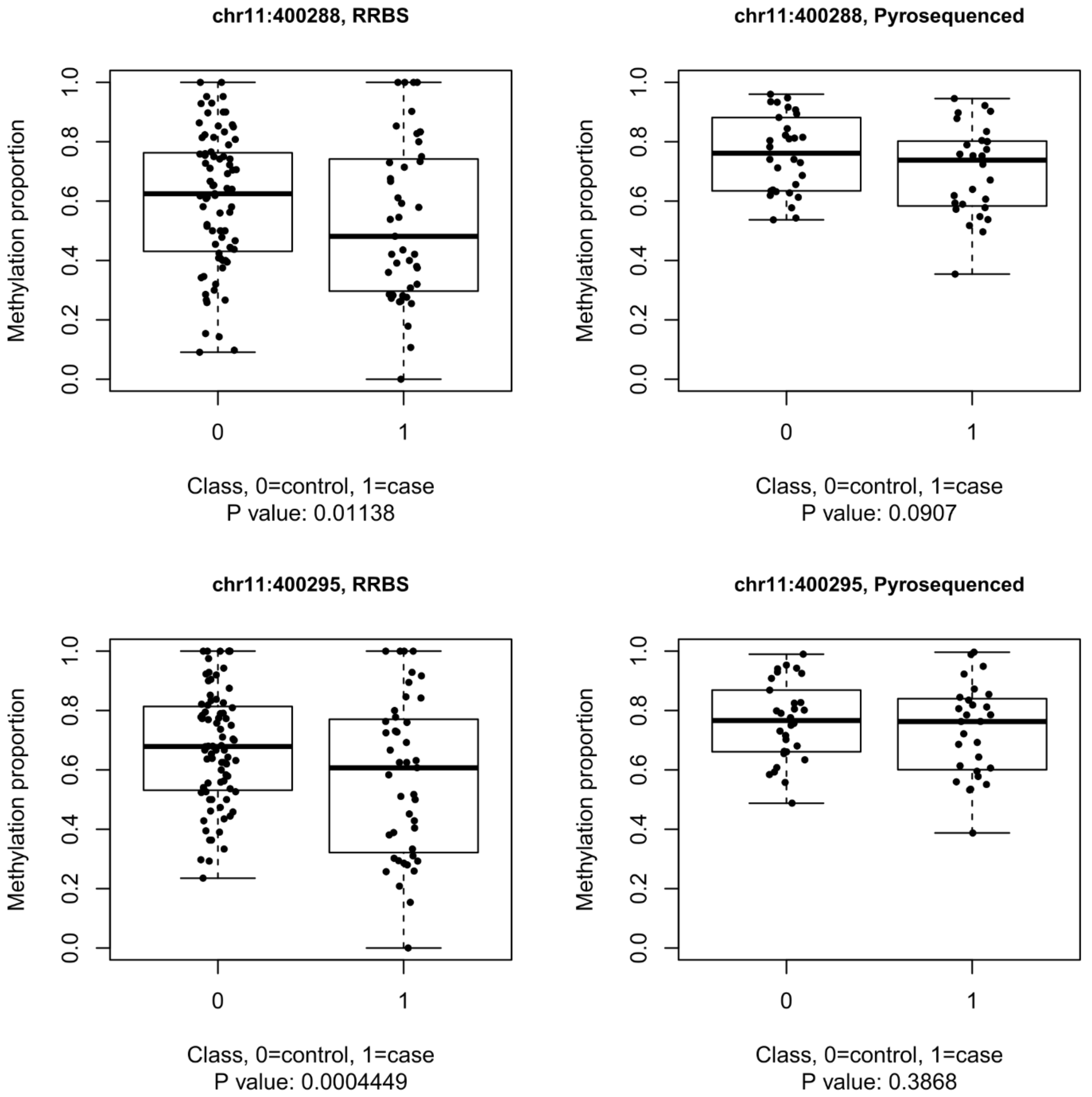

ESM Figure 5: Altogether 6 sex-associated differentially methylated cytosines on the promoter of Zona Pellucida Binding Protein 2 (*ZPBP2*) were technically validated by targeted pyrosequencing. The RRBS and pyrosequencing results are presented here as boxplots. The raw *p* values for differential methylation with respect to sex (based on GLMM for RRBS and ordinary linear model for pyrosequencing, separately at each CpG site) are marked below each plot. The midline of each boxplot is drawn at the median, boxes range from the 1st to the 3rd quartile, and whiskers extend to the most extreme values. These results have been described earlier in [2].

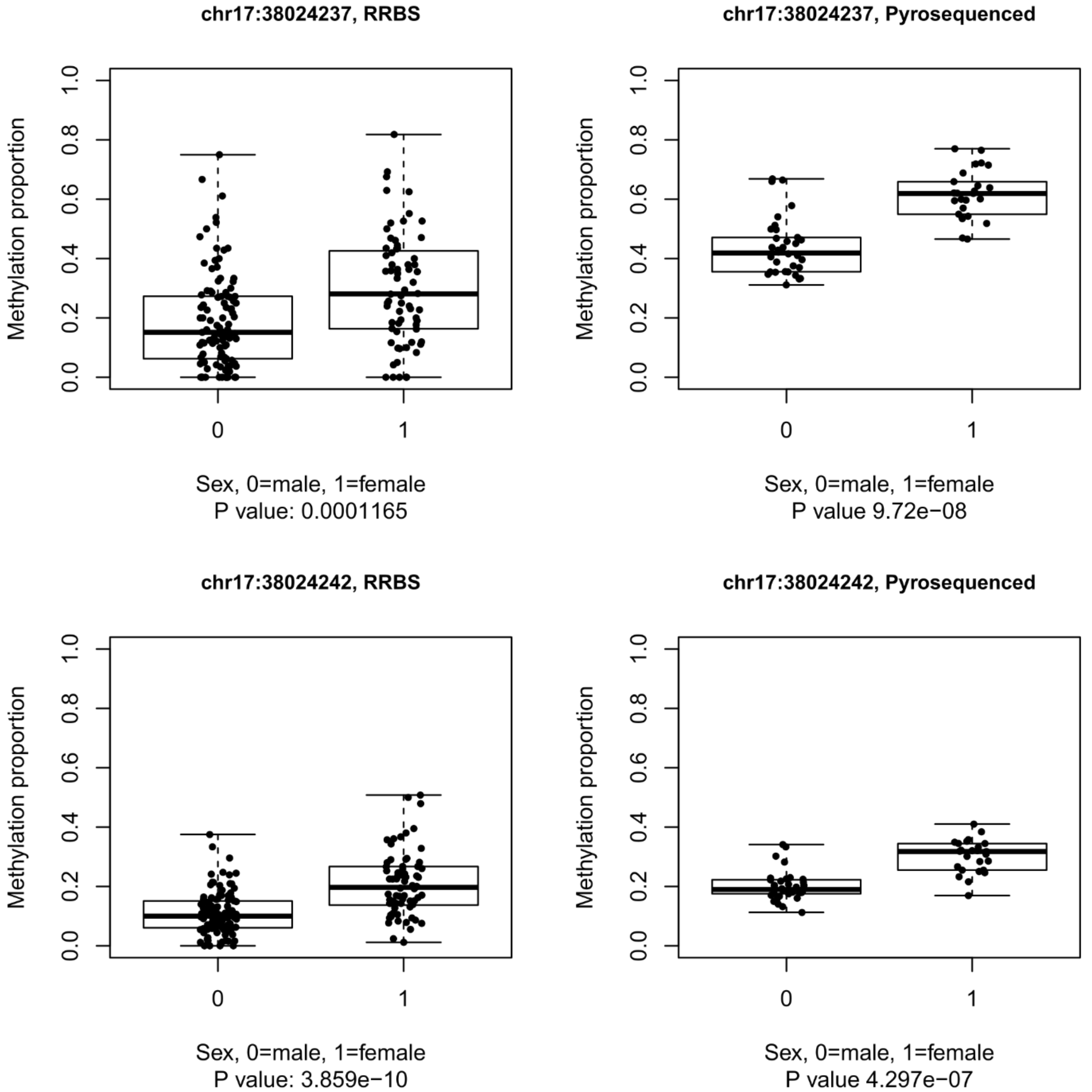

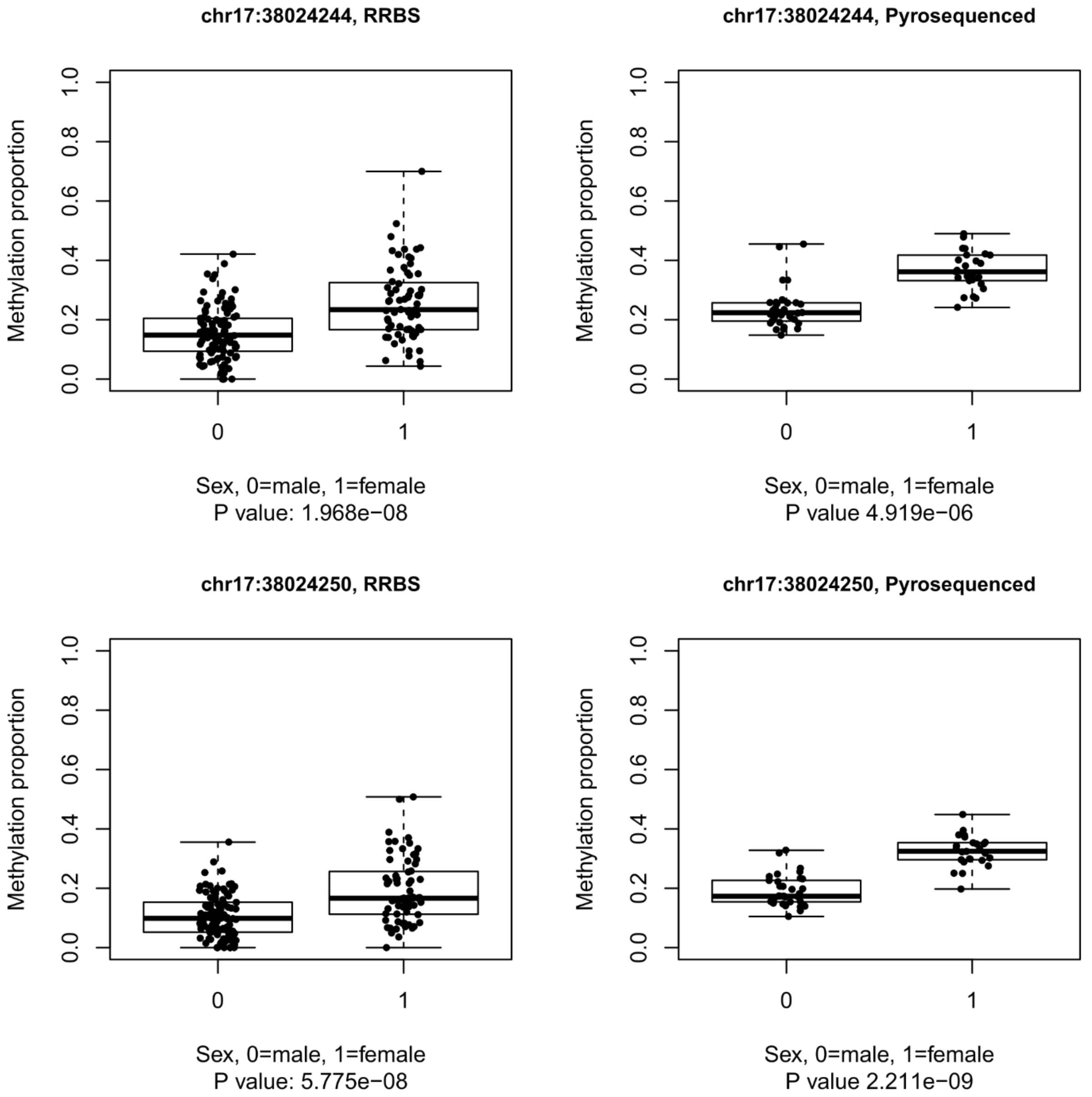
chr17:38024244, RRBS
Methylation proportion
0.0
0.2
0.4
0.6
0.8
1.0
0
1
Sex, 0=male, 1=female
P value: 1.968e−08
chr17:38024244, Pyrosequenced
Methylation proportion
0.0
0.2
0.4
0.6
0.8
1.0
0
1
Sex, 0=male, 1=female
P value 4.919e−06
chr17:38024250, RRBS
Methylation proportion
0.0
0.2
0.4
0.6
0.8
1.0
0
1
Sex, 0=male, 1=female
P value: 5.775e−08
chr17:38024250, Pyrosequenced
Methylation proportion
0.0
0.2
0.4
0.6
0.8
1.0
0
1
Sex, 0=male, 1=female
P value 2.211e−09

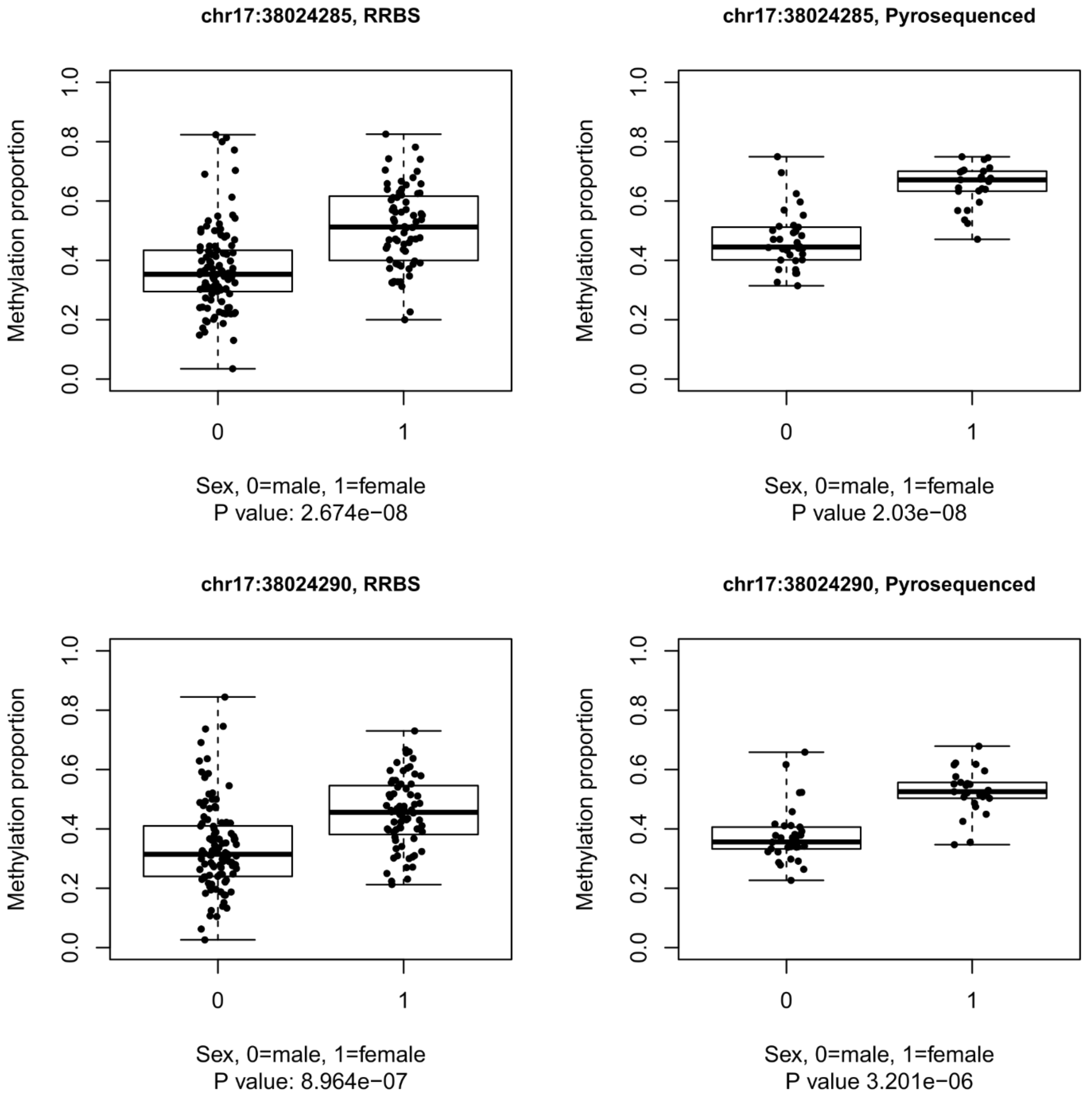
chr17:38024285, RRBS
Methylation proportion
0.0
0.2
0.4
0.6
0.8
1.0
0
1
Sex, 0=male, 1=female
P value: 2.674e−08
chr17:38024285, Pyrosequenced
Methylation proportion
0.0
0.2
0.4
0.6
0.8
1.0
0
1
Sex, 0=male, 1=female
P value 2.03e−08
chr17:38024290, RRBS
Methylation proportion
0.0
0.2
0.4
0.6
0.8
1.0
0
1
Sex, 0=male, 1=female
P value: 8.964e−07
chr17:38024290, Pyrosequenced
Methylation proportion
0.0
0.2
0.4
0.6
0.8
1.0
0
1
Sex, 0=male, 1=female
P value 3.201e−06
